# 

**Published:** 1914-04-25

**Authors:** 


### BEDS ENDOWED BY HOSPITAL FOUNDER.

We had recently to chronicle the death of Mr. H. J. Casey, to whom, practically speaking, was due the founding of the Walthamstow General Hospital. We now learn that under his will the sum of £6,000 has been left to the hospital to be used in the endowment of six beds in that institution in memory of Mr. and Mrs. Casey.

### THE RELATION OF LEGACY TO SUBSCRIPTIONS.

Mr. Septimus Brocklehurst, of Sefton Park, Liverpool, who left estate of which the net personalty has been sworn at £346,916, has bequeathed £1,000 each to the Liverpool Convalescent Institution, Woolton; the Bluecoat Hospital, Liverpool; the Royal Liverpool County (*sic*) Hospital for Children, Heswall; the Children's Convalescent Home, West Kirby; and the St. Paul's Eye Hospital and George Edward Walker Memorial, Liverpool. He left also to other charities to which he subscribed a legacy equal to ten times the amount of such subscription.

### Appointments.

Dr. Gordon Holmes, formerly physician to out-patients at the Seamen's Hospital, Greenwich, and lately elected physician to Charing Cross Hospital, has been appointed neurologist to the hospital at Greenwich, where the following new appointments have been made on the resident staff for the next six months:—House physicians: Dr. W. S. Lindsay, M.B., Ch.B. Edin., and Dr. D. McKelvey, M.B., Ch.B.; house surgeons: Mr. H. Tomlin Hunter, M.B., B.S. Durham, and John J. S. Rowe, L.M.S.S.A. The board of management have also appointed Mr. Geoffrey Hadfield, M.B., B.S., of St. Bartholomew's Hospital, to the post of medical registrar.

### ORDER OF ST. JOHN OF JERUSALEM.

The King has sanctioned the following among other promotions in and appointments to the Order of the Hospital of St. John of Jerusalem in England:—Knights of Grace: Colonel C. R. Tyrrell, M.R.C.S. (from Esquire); Mr. R. H. Grimbly, M.R.C.S. (from Honorary Associate). Lady of Grace: Mrs. G. E. Twiss (from Honorary Serving Sister).

**Doctors' Wives Association.**

(*See page* 87.)

**COUPON.**

I hereby certify that I am a doctor's wife, and I beg to send you my name and address as one who wishes to become a member of the proposed Doctors' Wives Association. I hereby agree to qualify as a member by paying the subscription of 7*s.* 6*d.* per annum on or before May 1, 1914, such subscription entitling me each year to a free copy of The Hospital newspaper delivered weekly, with the Doctors' Wives section, and to include my annual subscription to the Association.

This agreement undertaking to subscribe on my part is subject to at least 1,000 doctors' wives conditionally joining the proposed Association by signing and returning to "Co-operation" as below by April 18 next a similar coupon to that to which I hereby attach my signature.

(Signed) ..........................................

(Wife of) * ..........................................

(Address) ..........................................

..........................................

(Date) ..........................................

*Note.*—When filled in and signed cut out this coupon, place it in an envelope and address and post it to:

Co-Operation,
c/o The Scientific Press,
28 & 29 Southampton Street, Strand,
London, W.C.

* Here insert full name and qualifications.

### Gifts and Bequests.

The Rev. John Henry Ashley Gibson, of Brighton, has left £50 to the Brighton, Hove, and Preston General Dispensary.

The Eversfield Hospital for Consumption and Diseases of the Chest, St. Leonards-on-Sea, has received from the executors of the late Miss Evelyn Mary Eliza Russell, of Chenies House, Chenies, Bucks, the sum of £400 free of legacy duty. Miss Russell was a daughter of the Rev. Lord Wriothesley Russell, Canon of Windsor, Chaplain to her Majesty Queen Victoria, and granddaughter of the sixth Duke of Bedford.

Dame Sarah Annabella Boughey, of Shrewsbury, has bequeathed £15,000 for a cottage hospital at Newport.

The Treasurers of the Middlesex Hospital have received £250 from Sir John Glover through Sir Alfred Pearce Gould.

Colonel Charles John Bullivant Parker, of Grantham, Lancashire, banker, has left £200 each to the Grantham Cottage Hospital and the Victoria District Nursing Association, Grantham.

Mr. Robert Kelsey Hill, of Doncaster, retired chemist and druggist, has left £1,000 each to the Huddersfield Infirmary, the Dewsbury Infirmary, the Wakefield Infirmary, and the Doncaster Infirmary.

Mr. John Ritchie, of Liverpool, has bequeathed £250 each to the following Liverpool institutions:—The Royal Infirmary, the Royal Southern Hospital, the "David Lewis Northern Hospital," the Infirmary for Children, the Bluecoat Hospital, and the Bootle Borough Hospital.

Mr. Alfred Bradley, of Nottingham, has left £1,000 each to the Nottingham Day Nursery and Orphanage and the Nottingham General Hospital, £500 each to the Midland Counties Home for Incurables and the Nottingham and Notts Association for the Prevention of Consumption, and £1,500 to other charities.

Mr. Francis Arthur Farrell, of 37 Merrion Square, Dublin, has left £200 each to the Hospital for Incurables at Donnybrook; St. Mary's Asylum for the Female Blind, Merrion, Dublin; the Mater Misericordiæ Hospital; St Vincent's Hospital, Stephen's Green, Dublin; and St. Michael's Hospital, Kingstown.

Rates of Subscription: Twelve months, 6/6; Six months, 4/-; Three months, 2/-, post free to any address in the United Kingdom. Abroad, Twelve months, 8/8; Six months, 5/-; Three months, 2/6, post free.—Address The Subscription Dept., "The Hospital," The Hospital Building, 28/29 Southampton Street, Strand, London, W.C.

